# Association between mental health symptoms and behavioral performance in younger vs. older online workers

**DOI:** 10.3389/fpsyt.2023.995445

**Published:** 2023-03-29

**Authors:** Colleen Mills-Finnerty, Halee Staggs, Nichole Hogoboom, Sharon Naparstek, Tiffany Harvey, Sherry A. Beaudreau, Ruth O’Hara

**Affiliations:** ^1^Sierra Pacific Mental Illness, Research, Education, and Clinical Center, Veterans Administration Palo Alto Health Care System, Palo Alto, CA, United States; ^2^Department of Psychiatry and Behavioral Sciences, Stanford University, Palo Alto, CA, United States; ^3^Shiley-Marcos School of Engineering, University of San Diego, San Diego, CA, United States; ^4^Department of Psychology, San Jose State University, San Jose, CA, United States; ^5^Department of Psychology, Bar-Ilan University, Ramat Gan, Israel; ^6^Department of Biochemistry, University of Tennessee at Chattanooga, Chattanooga, TN, United States; ^7^School of Psychology, The University of Queensland, Brisbane, QLD, Australia

**Keywords:** COVID-19, depression, anxiety, behavior and cognition, computational modeling, Bayesian analysis

## Abstract

**Background:**

The COVID-19 pandemic has been associated with increased rates of mental health problems, particularly in younger people.

**Objective:**

We quantified mental health of online workers before and during the COVID-19 pandemic, and cognition during the early stages of the pandemic in 2020. A pre-registered data analysis plan was completed, testing the following three hypotheses: reward-related behaviors will remain intact as age increases; cognitive performance will decline with age; mood symptoms will worsen during the pandemic compared to before. We also conducted exploratory analyses including Bayesian computational modeling of latent cognitive parameters.

**Methods:**

Self-report depression (Patient Health Questionnaire 8) and anxiety (General Anxiety Disorder 7) prevalence were compared from two samples of Amazon Mechanical Turk (MTurk) workers ages 18–76: pre-COVID 2018 (*N* = 799) and peri-COVID 2020 (*N* = 233). The peri-COVID sample also completed a browser-based neurocognitive test battery.

**Results:**

We found support for two out of three pre-registered hypotheses. Notably our hypothesis that mental health symptoms would increase in the peri-COVID sample compared to pre-COVID sample was not supported: both groups reported high mental health burden, especially younger online workers. Higher mental health symptoms were associated with negative impacts on cognitive performance (speed/accuracy tradeoffs) in the peri-COVID sample. We found support for two hypotheses: reaction time slows down with age in two of three attention tasks tested, whereas reward function and accuracy appear to be preserved with age.

**Conclusion:**

This study identified high mental health burden, particularly in younger online workers, and associated negative impacts on cognitive function.

## Introduction

The global COVID-19 pandemic has had significant impacts on mental health. However, not all groups of people have been equally impacted. Younger people have reported higher rates of depression and anxiety (1, 2), substance abuse (3–5), and suicidality (3) since the beginning of the pandemic (6). Conversely, older adults have shown resilience in several large studies (2, 6–12). In this manuscript, we report results from a pre-registered study (13) of mental health and cognitive function in a diverse sample of online workers, collected before and during the COVID-19 pandemic. We show that there are significant disparities in mental health outcomes based on group demographics and extend previous work (14) by linking mental health symptoms to cognitive performance.

It is well established that in healthy people, mental health generally improves with age. This is often attributed to increased perception of control (15, 16), and attention toward positive stimuli toward the end of life, known as socioemotional selectivity theory (17, 18). This positivity effect reflects healthy engagement of pleasure and reward pathways in the brain. The integrity of these corticostriatal dopaminergic networks is essential for maintaining good mental health (19, 20). Indeed, even throughout the COVID-19 pandemic, older adults from multiple countries were at greater risk of contracting the virus, but generally have been found to have stable mental health and general wellbeing (1, 2, 7). Younger people have shown the opposite pattern (9, 11, 12). However, impacts of the pandemic on cognitive functioning in online workers remains under-characterized.

Understanding the mental health and cognitive status of the growing online workforce is timely, especially under persistent pandemic conditions necessitating wide adoption of long-term, work-from-home accommodations. Cognitive impairments are a symptom of depression (21) and neurodegenerative diseases such as dementia (22). Better understanding the cognitive performance of people working from home, in a cohort that ranges in age from 18 to 80, can aid in understanding the relationship between mood and cognition across the lifespan. For example, higher depression symptoms are associated with accelerated brain aging in mid-life, which is predictive of subsequent dementia diagnosis in later life (23, 24). Therefore, better understanding of mood-cognition relationships in a wide age range has relevance for brain health throughout the lifespan.

We preregistered our data collection and analysis plan to promote transparency, replicability, and reduce statistical degrees of freedom (13). The measures included self-report symptoms of anxiety (25), depression (26, 27), and wellbeing (28), as well as a short online neurocognitive task battery (see Study Design). Pre-registered hypotheses included the following: Hypothesis 1; reward-related behaviors will remain intact as age increases, specifically, consistently preferring immediate over delayed rewards in the Delay Discounting task (29–31), Hypothesis 2; cognition is hypothesized to decline with age, specifically resulting in slower performance on the Flanker (32–35), Visual Search (32–35), and Simon tasks (36–39), Hypothesis 3; depression (26, 27) and anxiety (25) symptoms will be higher for participants whose data is collected during the COVID-19 pandemic relative to a pre-pandemic baseline sample. We also report exploratory analyses linking latent cognitive variables to depression and anxiety symptoms.

## Materials and methods

### Preregistration

We designed our study in 2019 and preregistered the methods and hypotheses early in the COVID-19 pandemic. A pilot sample of *n* = 30 was collected, prior to preregistration using the Open Science Framework in March 2020 (13) to ascertain feasibility, quality check the data collection procedures, and assess attrition rates across the data collection platforms. Hypothesis testing for main effects of task conditions (manipulation check), and main effects of age on accuracy (validity check), were conducted with the pilot sample. The pilot data quality was found to meet criteria and was included in subsequent analyses.

**H1:** Reward-related behaviors will remain intact as age increases (i.e., no effect of age), including consistently preferring immediate over delayed rewards in the Delay Discounting task (29–31).

**H2:** Cognition is hypothesized to decline with age, specifically resulting in slower performance on the Flanker (32–35), Visual Search (32–35), and Simons tasks (36–38).

**H3:** Depression (26) and anxiety (25) symptoms will be higher for participants whose data is collected during the COVID-19 pandemic relative to a pre-pandemic comparison sample (14). The samples will be age-matched for 30 years and older to test this hypothesis.

### Instruments

#### Mental and physical health measures

The Patient Health Questionnaire-8 (PHQ8) (26) was given to the 2018 sample, and the Patient Health Questionnaire-9 (PHQ9) (27) was given to the 2020 sample. Both are clinical measures of self-reported depression symptoms on a 4-point Likert scale from 0 (*not at all*) to 3 (*nearly every day*). Item nine assesses suicidality and is not included in the PHQ8 version. For direct cohort comparisons, we use PHQ8 scoring in both groups. For analyses with only the 2020 cohort, we use PHQ9 scoring. The scores are highly correlated (*r* (212) = 1, *p* < 0.001), similar to past study findings (40–42). The Generalized Anxiety Disorder-7 (GAD7) (25) is a clinical measure of seven anxiety symptoms on a 4-point Likert scale from 0 (*not at all*) to 3 (*nearly every day*). Both versions of the PHQ and the GAD7 have been normed with clinical samples (25, 27). The total scores quantify symptom severity, with above five representing elevated symptoms, and above ten indicating clinically relevant severity (25, 27). In this study, we refer to scores of 5 and above as *suprathreshold*, and scores of 4 and below as *subthreshold*.

As specified in our pre-registration, we choose to characterize mood symptoms continuously rather than dichotomizing the data into normal control group versus mental health group, in order to improve detection power and to allow the data to reflect the naturally occurring distribution in the recruited sample of online workers.

#### Neurocognitive tasks

##### Delay discounting

Delay discounting (DD) is the decline in the present value of a reward with delay to its receipt. The DD procedure is widely used to study impulsivity across a range of populations and reward types (29–31, 43). It was chosen here due to its brevity as a behavioral measure of reward preferences, in order to test one of the key tenets of socioemotional selectivity theory (17): reward related behaviors do not decline with age. Impulsivity is operationalized as preferring immediate but smaller monetary rewards over delayed but larger monetary rewards. Participants were presented with 27 trials and chose either an immediate but smaller monetary reward (“Receive $20 now”) or a delayed but larger monetary reward (“Receive $55 in 7 days”). Responses were scored using an Excel-based, automatic, Monetary Choice Questionnaire Scorer (MCQ) which summarized multiple transformations of the K parameter (overall *K*, small, medium, large, and composite *K*), discount rates, consistency scores, and proportion measures for each participant (44). The possible range of *K* is 0 to infinity. Smaller *K*-values indicate a lack of discounting and a general preference for delayed rewards, whereas the larger values indicate heavy discounting and a preference for immediate rewards (i.e., higher impulsivity).

##### Simon task

The Simon task (36–38) presented the words “Left” and “Right” on the left and right sides of the screen, with the word either matching the position (e.g., “left” on left side; 20 congruent trials) or mismatching (e.g., “left” on the right side; 16 incongruent trials). Large black text was presented on a white background with a black fixation cross (600 ms) between trials. Trial order was randomized. We assessed reaction time, errors, and the effect of condition (congruent vs. incongruent).

##### Flanker

The Flanker task (32–35) involved a row of five cartoon, orange fish as stimuli against a white background. Participants were asked to watch the middle fish and hit the “F” key for swimming left, and “J” key for swimming right. The four fish surrounding the target were facing in the same direction as the target (congruent; 24 trials) or in the opposite direction (incongruent; 24 trials). A black fixation cross against a white background was presented for 400, 800, or 1,200 ms in between trials. Trial order was randomized, and four practice trials were given at the start. We assessed reaction time, errors, and the effect of condition (congruent vs. incongruent).

##### Visual search

The visual search task (32–35) required participants to locate an orange “L” among arrays of 9 to 15 distractors including orange and blue letters (“F” and “L”). The blue and orange letters were presented in large font against a white background, with a black fixation cross between trials (800 ms). Participants were instructed to press “F” if the orange “L” is present (15 trials), and press “J” if the orange “L” is absent (9 trials). Trial order was randomized. We assessed reaction time, errors, and the effect of task condition.

#### Remote data collection environment

Both samples completed REDCap surveys (45, 46), and the 2020 sample completed a custom task battery in Gorilla Experiment Builder (47) for browser-deployed cognitive testing. Participants first completed screening questions, followed by self-report measures for anxiety (25), depression (26, 27), general physical health (48, 49), and wellness (28) using REDCap (45, 46). Then, they were linked to a cognitive task battery in Gorilla (47) assessing the following domains: impulsivity (29), reward sensitivity (29), and visuospatial attention and interference (32, 35, 38). Participants responded to stimuli presented in a web browser and used keyboard responses. A narrative form catch question along with preregistered accuracy cutoffs were used to identify low quality data from participants (e.g., lack of English proficiency, bot auto-completers, or repeaters). The study took approximately 45 min to complete and was designed to be completed only once (participants who completed more than once were removed from analysis).

### Samples

**FIGURE 1:**
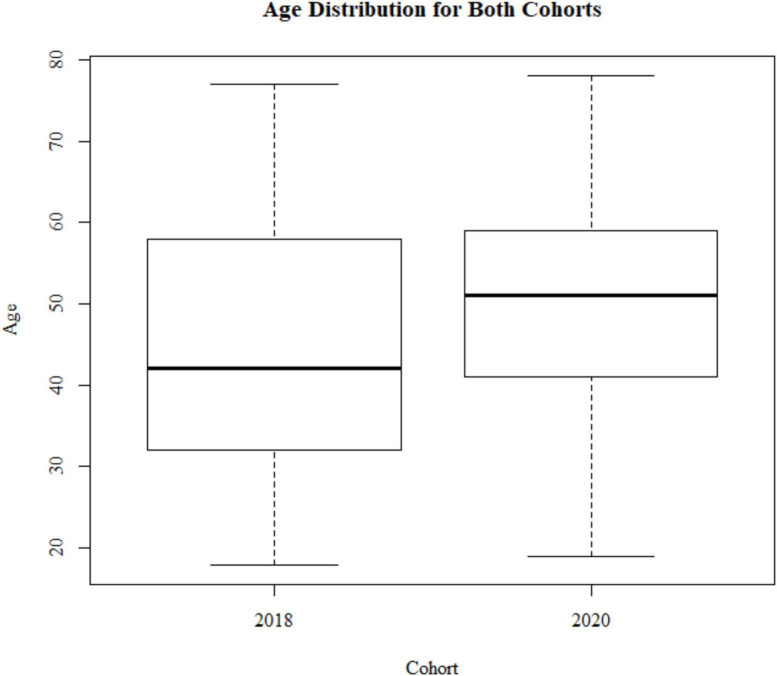
Sample demographics: age distribution for the 2018 cohort (*N* = 799) and 2020 cohort (*N* = 233).

**FIGURE 2:**
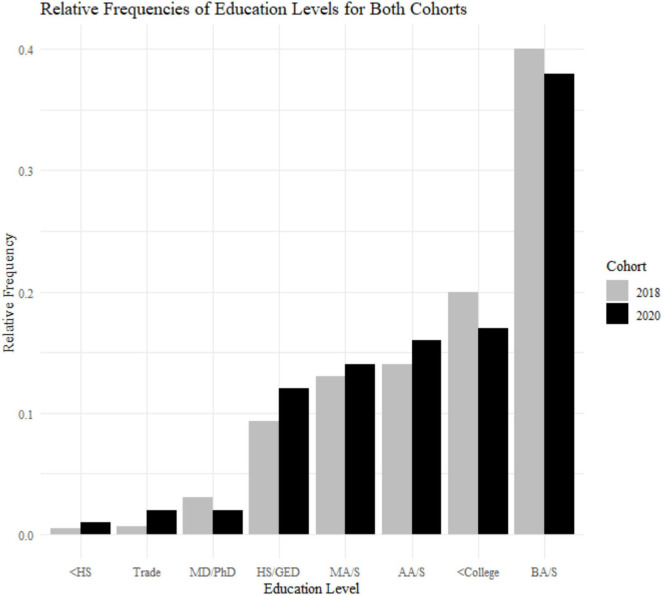
Sample demographics: education levels for the 2018 cohort (*N* = 799) and 2020 cohort (*N* = 233). <HS, less than high school; HS/GED, high school or general educational development; <College, some college but no degree; Trade, equivalent of trade or professional certificate; AA/S, Associate of Art or Science; BA/S, Bachelor of Art or Science; MA/S, Master of Art or Science; MD/PhD, doctoral level degree.

**FIGURE 3:**
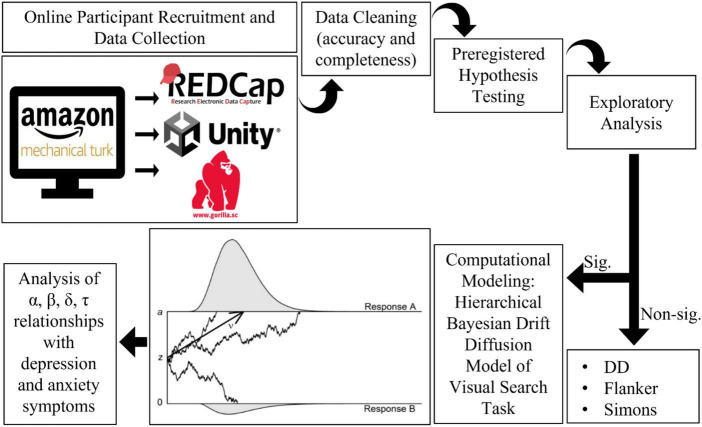
Study workflow.

Participants (*N* = 233) were recruited using Amazon MTurk (50) during a 4-week data collection period in Summer 2020. All participants were 18+, fluent in English, and had enough visual ability to complete the informed consent process and follow study instructions. Procedures and inclusion criteria match what were done for the comparison sample in 2018 (*N* = 799) (14). Our samples’ demographics (Figures 1–3) appear to resemble those found in other MTurk samples (51, 52). In a recent study, MTurk workers’ (*N* = 2,295) household income and household size were found to mirror the general population rates (52). Additionally, most people report MTurk income as paid leisure (56%) or a part-time job (37%), with almost 70% reporting that the income is used for non-essential expenses (52). Finally, 15% of workers reported having a disability, which is about half the amount of the general population (52).

Although COVID-19 forced a transition to work-from-home employment for many, Amazon MTurk workers (50) had already been used to the arrangement for some time. Online gig work through platforms such as MTurk provides short term employment opportunities that can be done on a laptop at home, such as transcription or research study participation. Researchers have benefited from having a ready-made participant pool to quickly and inexpensively collect large data sets. The MTurk workforce represents general population demographics, including income, race, and ethnicity (51, 52). However, there are slightly more American women working on MTurk than American men, and they are highly educated (51, 52).

### Sample descriptive statistics

A Levene test and Kruskal–Wallis test show that the two cohorts differ with respect to age variance and median [*F*(1) = 26.135, *p* < 0.001; *X*^2^(1) = 38.474, *p* < 0.001; Figure 1]. The 2020 cohort is older (mean age = 53.1, SD age = 10.2) compared to the 2018 cohort (mean age = 47.8, SD age = 12.6). This is due to the 2018 study having a minimum age of 18+, and the 2020 cohort having a minimum age of 30. Therefore, we used an age matched sample with a minimum of 30+ when comparing data between these two groups. The samples are different with respect to gender frequencies [*X*^2^(1) = 9.721, *p* = 0.0018; 2018, 52% female; 2020, 65% female]. There is no difference in education levels between the two cohorts [*X*^2^(7) = 5.678, *p* = 0.578; Figure 2].

#### Power calculations

Power calculations were based on effect size estimates derived from group differences in reaction times from a study using a similar behavioral paradigm (53), which had an effect size of Cohen *d* = 0.66. Note that the task domain that was the primary outcome variable at the time of preregistration was used to generate the estimates based on a within subjects test, however, analysis of that task is the subject of a separate manuscript as detailed in the section “Deviations from preregistration.” Power calculations for a within-subject one-tailed test with significance set to *P* = 0.05 identified that 30 participants were needed to achieve 80% power. Given the greater variability in cognitive function in older adults, exploratory aims of the study, and variable data quality and completeness resulting from online data collection, we aimed to collect 50 people for each age group (30–39, 40–49, 50–59, 60–69, 70–79, and 80+), acknowledging this was overambitious for people over 60 who have previously been less well represented on Amazon MTurk (14, 50). We aimed for the described age breakdown above, but those numbers were dependent on MTurk worker availability, attrition rates, and number of technical issues that impact study completion. We continued data collection until targets were met or exceeded, and the study budget was exhausted.

## Statistical models

Statistical analyses and data visualization were completed using R version 4.0.5 (54) and the following packages: lme4 (55), hBayesDM (56), ggplot2 (57), ggpubr (58), sjPlot version 2.8.9 (59), and stats (54). Linear mixed-effect models (critical *p*-value of.05) were used to quantify the main effect of age on the reward, attention, and health variables to test primary hypotheses: depression (26, 27) and anxiety (25) symptoms, reaction time, and accuracy in behavior tasks as applicable. Generalized linear models (GLMs) with Gaussian links were used in cases where data did not conform to assumptions of normal distribution. They were used to quantify individual differences in the relationship between age and *K*-values (log geomean transformed) (29, 44). In exploratory analysis, GLMs with Poisson links were used to quantify individual differences in mood disorder symptoms and cognitive function.

### Computational modeling

Hierarchical Bayesian drift diffusion modeling was used to generate latent cognitive parameters (60–62). Briefly, the hBayesDM package in R (56) uses a Markov Chain Monte-Carlo sampler (63) to generate a posterior distribution of parameter values seeded using the actual participant data. These distributions are used as a hyperparameter to reduce measurement error (64). In two-choice decision tasks, this improves estimation accuracy and robustness to outliers (60, 61). The model quantifies four parameters for each participant: alpha, beta, tau, and delta. Tau represents non-decision time, which is the shortest valid reaction time (excluding false positives) representing the minimum motor preparation needed before a response (button press) can occur (65). Alpha is the decision boundary, or, the amount of evidence needed to make a decision (65). Greater boundary values suggest more information is needed; having a lower boundary value indicates less information is needed. Beta represents the bias to respond, such as if participants tend to respond more to stimuli on the right side of the screen relative to the left (65). Delta, or drift rate, quantifies the rate of evidence accumulation–the speed at which the person can integrate information in favor of a decision (65).

### Data exclusion

Data for this study was not excluded based on symptom or health scores. We included as much data as possible since slower task performance was hypothesized for older participants. Data with less than 70% accuracy was reviewed, and data with accuracy below chance (less than 50%) was excluded. Linear mixed-effects models allow for partially incomplete data. If a participant completed at least one-third of the battery, they were included in the respective task sub-sample.

### Deviations from preregistration

The analysis plan outlined in the preregistration was followed with the exception of: excluding individual outlier trials for participants (instead using overall accuracy to minimize data loss), and the analysis of value-based attention capture (VBAC) and Willingness to Pay (WTP) data will be the subject of a separate manuscript.

## Results

### Analysis workflow

See Figure 3 for an overview of the analysis workflow. All *p*-values are reported raw and with a False Discovery Rate correction for all preregistered hypotheses (66). FDR adjusted *p*-values are not reported for exploratory analyses (66).

### Planned analyses

Hypothesis 1

We confirmed hypothesis 1: reward-related behaviors remained intact as age increases, including consistently preferring immediate over delayed rewards in the Delay Discounting task (29). Age did not have a linear relationship with the delayed discounting variables (Table 1).

**TABLE 1:** Additional analysis of delay discounting parameters and age, using linear mixed-effect models for delayed discounting parameters in the 2020 cohort (*n* = 207).

DD parameter	Formula	Variable			
		***t***	***df***	***p***	***Adj.***
Consistency	Age∼Consistency	0.47	207	0.639	1.00
Proportion	Age∼Proportion	1.22	207	0.225	0.675

Hypothesis 2

We partially confirmed hypothesis 2: cognition declined with age, specifically resulting in slower performance on the Visual Search (35) and Simons tasks (38), but not the Flanker (32). Age was not associated with changes in accuracy (Table 2). Age has a positive, linear relationship with reaction time for the Visual Search and Simons task (Table 3).

**TABLE 2:** Age is not associated with accuracy.

Task	Cond	Cohort 2020	Mean score	Model	Predictor			
		***n***	**(SD)**		***t***	***df***	***p***	***Adj.***
Simons	Both	195	0.96 (0.19)	Accuracy∼Age	0	194	1	1.00
VS	Pres	158	0.87 (0.12)	Accuracy∼Age	0	156	0.99	1.00
VS	Abs	158	0.95 (0.09)	Accuracy∼Age	0	156	0.99	1.00
Flanker	Both	139	0.99 (0.03)	Accuracy∼Age	0	204	1	1.00

Cond, condition; pres, present; abs, absent.

**TABLE 3:** Results of the pre-registered data analysis plan.

Hypotheses	Condition		2018		2020		Test	Formula		Coefficients			Supported
			**Score**		**Score**	**RT**							
		***n***	**mean(SD)**	***n***	**mean (SD)**	**mean (SD)**			***t/z***	***df***	***P***	**FDR**	
**Delay Discounting**														
1a: *K*-Values	–	–	–	207	–	–	GLM	Age∼K_log_geomean	−1.37	207	0.173	0.3	Yes
2a: Simons	Both	–	–	195	–	–	LMEM	RT_log∼Condition	15.2	194	<0.001	0.24E-17	Yes
	Con	–	–	–	0.96 (0.12)	0.55 (0.06) s	LMEM	RT_log_mn∼Age	3.22	194	0.0026	0.006	Yes
	Incon	–	–	–	0.84 (0.18)	0.60 (0.06) s	LMEM	RT_log_mn∼Age	4.13	194	<0.001	5.07E-5	Yes
2b: Visual Search	Both	–	–	158	–	–	LMEM	RT∼Condition	3.4	157	<0.001	0.02	Yes
	Con	–	–	–	–	1.3 (0.73)	LMEM	Age∼RT_log_mn	3.88	156	<0.001	0.013	Yes
	Incon	–	–	–	–	1.6 (1.3)	LMEM	Age∼RT_log_mn	2.88	156	0.0046	0.05	Yes
2c: Flanker	Both	–	–	139	–	–	Lmem	RT_log∼Condition	4.89	204	<0.001	0.005	Yes
	Con	–	–	–	–	0.57 (0.14)	LMEM	Age∼RT_log_mn	0.29	204	0.775	0.93	No
	Incon	–	–	–	–	0.58 (0.13) s	LMEM	Age∼RT_log_mn	0.33	204	0.738	0.98	No
3a: PHQ-8	–	626	4.98 (4.91)	211	4.27 (4.74)	-	*t*-test	PHQ∼Cohort	−1.879	370.3	0.0611	0.12	No
3b: GAD-7	–	605	4.08 (4.76)	211	3.92 (4.56)	-	*t*-test	GAD∼Cohort	−0.44	377.03	0.660	0.99	No

Mood measure *t*-tests are age-matched 30 years and older. Con, congruent; incon, incongruent; GLM, generalized linear model; LMEM, linear mixed effects model; RT, reaction time; log_mn, log transformed mean.

Hypothesis 3

We did not confirm hypothesis 3: depression (26) symptoms and anxiety (25) symptoms did not significantly differ between samples (Table 3).

### Age and mental health scores

#### Mood symptom correlations pre- and peri-COVID

**FIGURE 4:**
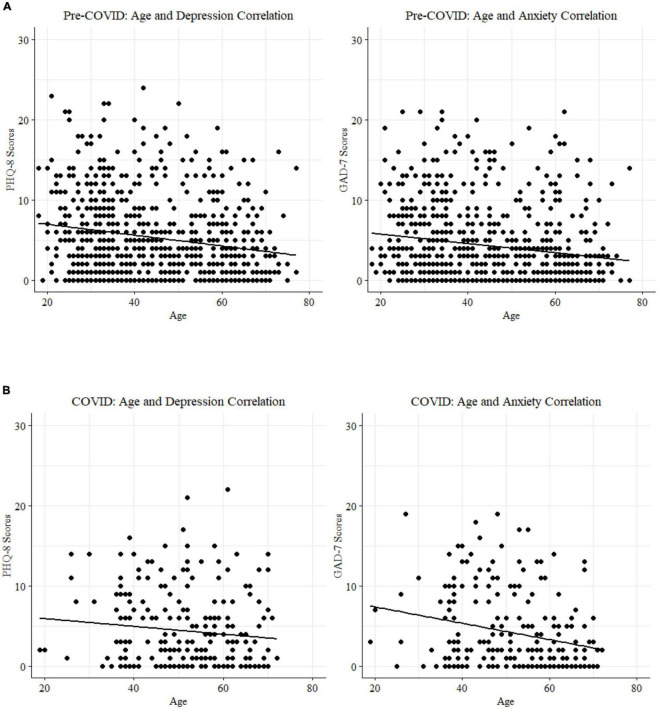
**(A)** Age and mood score correlations for the pre-COVID 2018 cohort. **(B)** Age and mood score correlations for the peri-COVID 2020 cohort.

Statistically significant negative correlations (Spearman *r*; Table 4) between age and anxiety were replicated in both the 2020 sample (*N* = 233; Figure 4A) and the pre-COVID 2018 sample (*n* = 740 PHQ8 (26); *n* = 718 GAD7 (25); Figure 4B). Splitting the samples at the age-matched median (53 years) for visualization purposes highlights the elevated self-reported depression and anxiety symptoms in the younger half of participants from both cohorts. Two-sample tests for equality of proportions (all *n* > 26) were statistically significant for suprathreshold scores (> = 5) between younger and older participants in both samples for anxiety symptoms (2018, *p* < 0.001; 2020, *p* = 0.0013) and depression symptoms (*p* < 0.001 for both cohorts).

**TABLE 4:** Correlations of age and mental health scores for each cohort.

Cohort	Measure	Coefficients		
		***r***	***df***	***p***
2018	GAD7	−0.2	716	<0.001
	PHQ8	−0.18	738	<0.001
2020	GAD7	−0.24	231	<0.001
	PHQ8	−0.12	231	0.0743

#### Computational modeling

To reduce experimenter degrees of freedom, and increase confidence in exploratory hypothesis generating results, the following workflow logic was used to identify data for computational modeling: (1) hypothesized main effect of condition was observed, (2) hypothesized age-related reaction time slowing was observed, and (3) a statistically significant relationship between mental health and task performance was observed. Data was entered into the hBayesDM (56) model for generation of latent cognitive parameters (α–alpha, β–beta, δ–delta, and τ–tau) which were used in further analysis of mood-behavior and mood-age-behavior relationships. The only task that met these criteria was Visual Search (34, 35). We did not consider the Flanker for exploratory analysis since our original hypothesized RT∼age relationship was not confirmed and this data didn’t meet criteria for further analysis. Analysis of mood symptom-behavior relationships for the Simons task data found no significant association between PHQ9 and reaction time in the Incongruent (*p* = 0.2) or Congruent condition (*p* = 0.09) and no association between GAD7 and reaction time in Incongruent (*p* = 0.9) or Congruent condition (*p* = 0.6).

#### HDDM of visual search

The absent (*n* = 172) and present (*n* = 169) conditions of Visual Search showed statistically significant differences in parameter means for all four drift diffusion model parameters: alpha (decision boundary), beta (bias), delta (drift rate), and tau (non-decision time) using a two-tailed *t*-tests (Figure 3 and Table 5).

**TABLE 5:** Results of cognitive parameter *t*-tests for Visual Search conditions.

Parameter	Condition	Mean	Coefficients		
			***t***	***df***	***p***
alpha, α	Absent	2.45	−7.539	324	<0.001
	Present	2.09			
beta, β	Absent	0.41	19.93	336	<0.001
	Present	0.604			
delta, δ	Absent	-1.249	39.259	339	<0.001
	Present	0.897			
tau, τ	Absent	0.69	–7.236	292	<0.001
	Present	0.55			

#### Separable behavioral phenotypes for anxiety vs. depression

Generalized linear models identified relationships between mood symptoms and latent cognitive parameters in the incongruent condition (*n* = 172) of the Visual Search task (Table 6). Participants higher in anxiety were faster to respond (lower tau/shorter non-decision times), but less accurate, and incorporated less information when making decisions (lower alpha/decision boundary). Participants higher in depression were slower to accumulate evidence in favor of a decision (higher delta/drift rate), and were more biased toward a particular response (beta parameter).

**TABLE 6:** GLMs for the association between cognitive parameters and symptom scores.

Outcome	Variables	Coefficients		
		***z***	***df***	***p***
GAD7	alpha, α	-2.747	170	0.00601
GAD7	beta, β	-1.353	170	0.176
GAD7	delta, δ	1.741	170	0.0818
GAD7	tau, τ	-4.454	170	<0.001
GAD7	accuracy	-4.361	170	<0.001
PHQ9	alpha, α	0.62	170	0.535
PHQ9	beta, β	-2.751	170	0.00594
PHQ9	delta, δ	2.521	170	0.0117
PHQ9	tau, τ	-2.335	170	0.0196
PHQ9	accuracy	-1.874	170	0.0609

#### Latent cognitive parameters are associated with anxiety and depression, accounting for age

**FIGURE 5:**
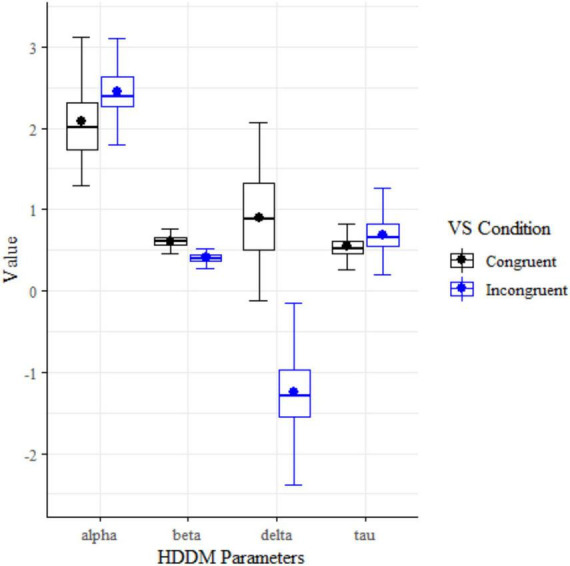
HDDM cognitive parameter box plots for Visual Search conditions.

**FIGURE 6:**
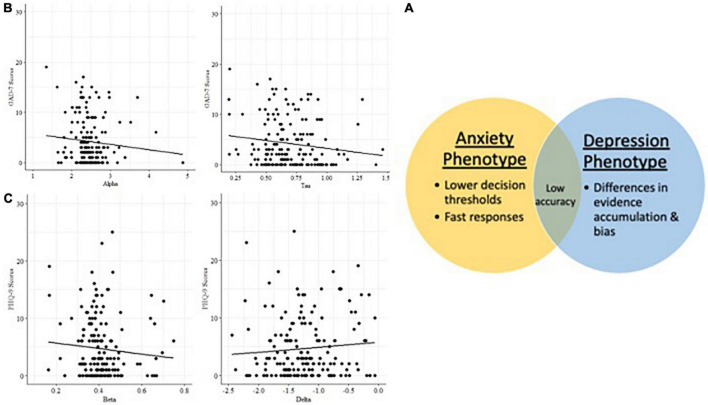
**(A)** Latent cognitive variables associated with anxiety and depression phenotypes in Visual Search. **(B)** Anxiety phenotype: decision boundary and non-decision time. **(C)** Depression phenotype: bias and drift rate.

**FIGURE 7:**
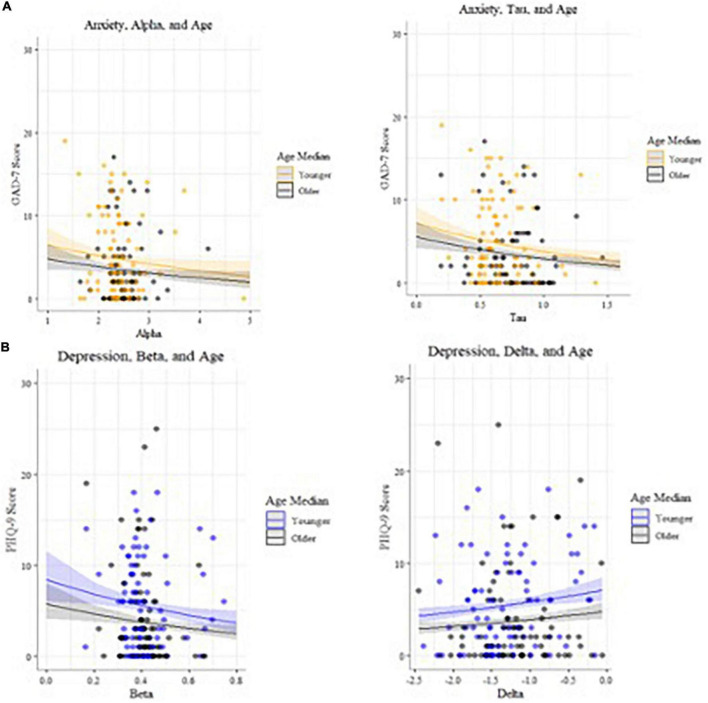
**(A)** Models of anxiety predictors, plotted by age (median split used for visualization purposes). **(B)** Models of depression predictors, plotted by age (median split used for visualization purposes).

Alpha (decision boundary) and tau (non-decision time) parameters in the incongruent condition are associated with higher GAD7 (25) scores (Figure 5), with age included in the GLM (Table 7). Decision boundaries were lower, and non-decision times were shorter, in those with higher anxiety. Beta (bias) and delta (drift rate) are associated with higher PHQ9 (27) scores (Figure 5); those higher in depression showed more biased responses, and a slower rate of evidence accumulation (drift rate). Age also independently predicted anxiety scores, with older age associated with lower severity of anxiety and depressive symptoms. To aid interpretation of the significant results for interactions between age and task accuracy, and age and computational modeling parameters, we utilized a median split of age for visualization purposes in Figures 5–7.

**TABLE 7:** Generalized linear models predict mood scores when accounting for age.

Outcome	Variables	Coefficients		
		***z***	***df***	***p***
GAD7	alpha, α	-2.034	169	0.042
	age	-8.111	169	<0.001
GAD7	tau, τ	-2.477	169	0.0132
	age	-7.439	169	<0.001
PHQ9	beta, β	-2.4	169	0.0164
	age	-7.808	169	<0.001
PHQ9	delta, δ	2.596	169	<0.001
	age	-7.959	169	<0.001

#### Age and accuracy scores are associated with mood symptoms

**FIGURE 8:**
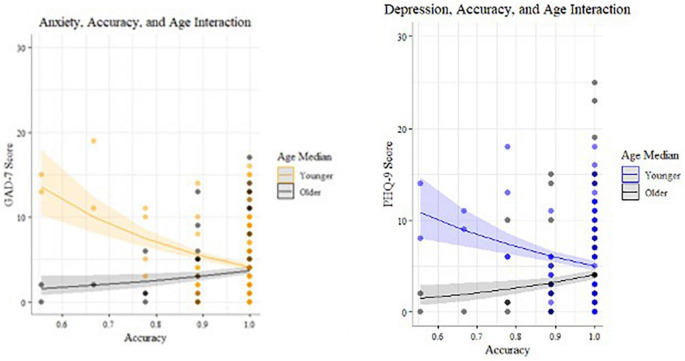
Statistically significant interactions predicting anxiety and depression for incongruent Visual Search (median split by age for visualization purposes).

In the incongruent condition (*n* = 172), there were statistically significant interactions (Table 8) between age and accuracy that predicted both depression (27) and anxiety (25) symptom scores (Figures 6, 8). Younger adults with higher anxiety and depression symptoms had lower accuracy. Age was split at the median (53 years) for visualization purposes.

**TABLE 8:** Generalized linear models of mood scores and associated interactions for Visual Search conditions.

Outcome	Condition	Variables	Coefficients		
			***z***	***df***	***p***
GAD7	Incon	accuracy × age	4.058	168	<0.001
PHQ9	Incon	accuracy × age	3.257	168	0.00113

#### Age and sex are associated with mood

In the 2020 pandemic sub-sample with sex data (*n* = 202, *n* = 137 female), GLMs show that sex is associated with mood symptoms, with women reporting higher anxiety (*p* = 0.017) and depression (*p* < 0.001) scores than men, when accounting for age.

#### Anxiety and depression comorbidity

**FIGURE 9:**
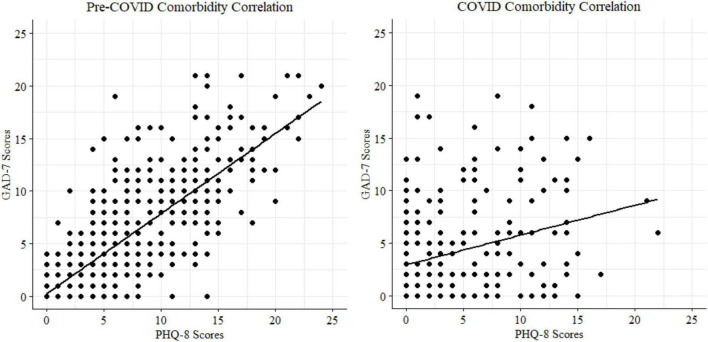
Cohort correlations between depression and anxiety scores.

Statistically significant positive correlations (Spearman *r*; Figure 9) were found between depression and anxiety symptom scores for the 2018 pre-pandemic cohort [*n* = 716 with both PHQ8 and GAD7 datapoints; *r* (699) = 0.81, *p* < 0.001] and the 2020 pandemic cohort [*N* = 233; *r* (231) = 0.31, *p* < 0.001].

## Discussion

This study examined mental health symptoms collected from over 1,200 online workers before and during the COVID-19 pandemic, as well as relationships between mood symptoms and cognition in the subsample collected during the pandemic. We found support for the majority of our hypotheses, including age-related slowing in response times and preserved reward function in older participants. The results replicated the mood-age interactions found in other pandemic samples (1, 7, 9, 11, 12), whereby older age is associated with lower mental health symptoms. However, analyses revealed an unexpected set of interactions: younger MTurk workers report higher mental health symptoms than older adults in the sample, which in turn negatively impacts their cognitive performance.

Perhaps the most notable finding was that compared to a pre-COVID, age-matched reference sample, there was not an increase in overall mental health symptoms (depression and anxiety) during the pandemic. This did not support hypothesis 3. Stress levels worldwide have been high during this pandemic, though lower rates have been reported in older adults in recent and past studies (2, 6, 9, 12). This resiliency may be due to higher pre-existing levels of social isolation in older adults, resulting in pandemic stay-at-home orders having a smaller impact. Social isolation may benefit older individuals because of reduced exposure to disease. Since our samples skewed older by design, this may explain the lack of difference. However, it is important to note that summer 2020 represented an early stage of the pandemic when mental health effects might not yet have become apparent in all groups. Some studies do report increases in symptoms, but trajectories differ based on demographics like gender or pre-existing health conditions (67, 68). For example, those who had a mental health diagnosis pre-pandemic were more likely to report stable mental health than those with no prior diagnoses. It is possible the already-elevated mental health symptoms in the Mturk workers we studied align with the latter set of findings. Self-reported symptoms of anxiety and depression were high in both the pre- and peri-pandemic samples (∼15–20% of the sample reporting moderate symptoms at clinical cut-offs), suggesting that online workers may generally experience higher mental health burden in a consistent manner.

Hypotheses 1 was supported, and Hypothesis 2 was partially supported (age related slowing for 2 of 3 attention tasks). We observed no effect of age on reward-related function [delay discounting task (29)]. We found statistically significant main effects of task condition: responses in the incongruent were slower than in the congruent condition, and older age was associated with slower responses during the Simons (37) and Visual Search tasks (35). These results replicate previous work showing age-related slowing during cognitive tasks in older adults (69), but preserved reward-related function (17), suggesting the data from our MTurk workforce participants bears reasonable resemblance to that of in-person lab studies (70). We did not find age related reaction time slowing for the Flanker test, possibly due to high accuracy rates/ceiling effects. The results showing no age effect on impulsivity run somewhat counter to other studies that have found changes in decision making throughout the lifespan (71), but are consistent with predictions based on socioemotional selectivity theory (17), specifically that intact reward function may be a feature of stable mental health in older adults (16).

Exploratory analysis identified a mental health-behavior relationship in the Visual Search task. There was a significant relationship between reaction time speed in the incongruent condition and anxiety symptoms, with those higher in anxiety exhibiting faster responses. Data were further analyzed using Hierarchical Bayesian Drift Diffusion modeling (56) to generate latent cognitive parameters. This analysis revealed statistically significant dissociations between symptoms of depression and anxiety. Participants higher in anxiety were faster to respond and incorporated less information when making decisions. Participants higher in depression, on the other hand, were slower to accumulate evidence in favor of a decision and more biased toward a particular response. Higher bias indicates a tendency to log the same response repetitively. It is defined for each individual based on the proportions of their responses, and therefore, at the group level, represents potentially different types of biases (e.g., a bias toward right button response and left button response cannot be differentiated). It is more analogous to the idea of “choice stickiness” and may represent deficits in set switching, specifically trouble adapting responses between congruent and incongruent trials, such as tending to over-respond that the stimulus is present when it is absent. Both depression and anxiety symptoms were related to reductions in accuracy.

These results are consistent with models of anxiety that posit that it may impact speed-accuracy tradeoffs in a manner following the Yerkes–Dodson arousal curve (72): subclinical anxiety may benefit performance, but higher anxiety decreases it. Our results showed those higher in anxiety had impairments in speed-accuracy tradeoffs, specifically shorter non-decision times, lower thresholds for making a decision (43), and less accurate decisions. The reasons for this finding deserve further study, but may be related to resolution of ambiguity aversion given that difficulty tolerating uncertainty is a vulnerability for mental health disorders, and particularly associated with anxiety (73). People with higher anxiety symptoms are more likely to interpret neutral stimuli as negative, and dislike scenarios that are more open-ended and ambiguous (73). The incongruent Visual Search condition is akin to searching for a familiar face in a crowd–one must decide when the search has been exhaustive enough to give up. This open-ended scenario may be more stressful for those with anxiety and lead to them rushing through trials, endorsing more false positives, or using guessing strategies to get through the task faster.

Conversely, higher depression symptoms were related to a pattern of responding involving lower accuracy, greater bias in responses, and slower evidence accumulation. Unlike the pattern seen with anxiety (faster/inaccurate), those higher in depression took longer to search the arrays for the missing target (accumulate evidence) in the incongruent condition. This is consistent with psychomotor slowing found in depression which is thought to be modulated by changes in dopaminergic and vascular function (19, 20, 74). These results deserve further study but suggest that in contrast to those higher in anxiety who may rush through the task, people with depression take longer to complete it because they spend more time searching for the missing target stimulus in the incongruent condition. Depression may reduce cognitive resources leading to less efficient information uptake, *via* impacts on fatigue, cognitive abilities, and motivation. It is not clear if people higher in depression symptoms were less capable of searching efficiently, or if they chose to take longer to search (e.g., lower motivation to complete the task efficiently), but either way it did not benefit their accuracy.

Interactions between age, mental health, and cognition were found. Younger participants reported greater mental health symptoms that negatively impacted cognition in a linear manner. This finding is notable given the recent evidence in several large scale samples that older age may be a protective factor against experiencing increased mental health symptoms during COVID (1, 7, 9, 11, 12). This study adds to this understanding by including objective behavioral evidence of cognitive dysfunction (reduced accuracy) in younger versus older adults suffering from mood symptoms. Of the participants endorsing clinically significant anxiety and depression (PHQ8 > = 10, GAD7 > = 10), two out of three were younger adults and one-third were older adults. Older adults reported lower levels of anxiety and depression on average (<5 on PHQ9 and GAD7), and those reporting slightly higher symptoms had slightly better accuracy than those with lower symptoms. These results may seem counterintuitive, however, note that the mean was higher and variance greater in younger adults. These findings suggest that subthreshold mental health symptoms may confer a benefit on some cognitive tasks in older adults, consistent with at least one study finding positive relationships between anxiety and task performance, possibly mediated by worry (75). Similarly, mild depression symptoms may benefit cognitive performance particularly in older adults; other research has shown that people who exhibit “depressive realism” tend to estimate outcomes less optimistically and therefore more accurately than those without depression (76, 77).

Finally, we observed substantial comorbidity between anxiety and depression (Spearman *r* of 0.81 and 0.31 for the pre- and peri-COVID samples, respectively). Model comparisons indicated that anxiety symptoms accounted for significant variance in depression scores and vice versa to similar degrees, even when accounting for age, task accuracy, and age/accuracy interactions. These findings are consistent with the high comorbidity between anxiety and depression in prior literature (78). Despite this high comorbidity, depression and anxiety symptoms were related to distinct latent cognitive parameters, suggesting that they are dissociable behaviorally.

Taken together these results reveal a dose-dependent relationship of mental health symptoms on cognitive performance, as well as age-related differences in mental health burden with significant impacts on cognitive function in younger adults.

### Limitations

There are strengths and limitations to using Amazon MTurk for data collection. First, data quality control requires extra time and care when participants complete the study in unknown conditions. To mitigate these impacts, we followed pre-registered criteria for exclusions based on accuracy, resulting in removal of approximately 15–30% of each tasks’ dataset before analysis. Additionally, a narrative form question was used to screen for auto-completer bots and participants lacking proficiency in English. Finally, we removed datasets with a high rate of “false positive” button presses (<2 ms) before conducting computational modeling analysis. We utilized Bayesian methods that are more accurate and robust to outliers to further increase the reliability of our findings (60).

Cohort effects may drive the age-related differences in mood symptoms and deserve further detailed study. For example, older MTurk workers may be higher in technical proficiency than community dwelling peer samples. This may be because the MTurk workforce is more educated than the general population (51, 52). They may be closer to a “super-ager” population who engage in online work for extra income and mental stimulation (79). They may also benefit from greater economic stability during their retirement from full time work, compared to younger Mturk workers. It is also possible older MTurk workers under-report mental health symptoms due to generational differences in stigma or age-specific stress factors. Such cohort effects likely contribute to some degree to the reported disparities in mental health symptoms. However, the suprathreshold symptom counts in younger adults show a clearly negative impact on cognition: higher errors, worse speed-accuracy tradeoffs, and slow, inaccurate performance.

A potential strength of our approach is access to a geographically and demographically diverse sample (relative to student convenience samples which are unlikely to be representative of the national US population). Participants completed the study in naturalistic conditions that reflect their typical work environment, and this may result in more accurate reporting of mental health symptoms due to the lack of study demand characteristics, such as a researcher observing their behavior (80). However, note that gig economy workers such as those on Amazon MTurk, likely differ from other people working from home in more traditionally hierarchical employment, even if they match on other demographic factors such as income and education. Additionally, we generally saw higher symptom self-report than typically found in the general population, which requires additional effort for participants to report (e.g., choosing between different symptom frequencies, vs. expending minimal effort by reporting no symptoms at all). Participants have no opportunity to gain monetarily or otherwise from reporting greater symptoms. The data also replicate the results from larger samples of online and younger workers (51, 52), suggesting the self-reports are likely to be accurate. However, the demand characteristics of teleresearch settings deserve future study (80).

Additionally, the high accuracy in most task conditions suggests that the sensitivity to detect effects of mental health on task performance may have been impacted by ceiling effects. The significant results between behavior and mood when examining the Visual Search task (35) may be related to increased difficulty producing a greater range of behavior (e.g., more false positives). Future studies could use adaptive versions of the cognitive tasks to see whether this improves sensitivity to detect mood effects on cognition.

Finally, while we confirmed hypothesis 1 using the preregistered criteria of failing to reject the null while testing the age-delay discounting relationship, this is not to be considered the same as evidence in favor of the null. A more robust approach future studies could take might include a Bayes Factor to estimate the support for the null (81, 82).

### Future directions

Future studies could use remote data collection of objective brain activity using an EEG array (83, 84), have participants undergo brain stimulation using a device such as transcutaneous vagus nerve stimulation (85) or transcranial direct current stimulation, or undergo fMRI scanning (86) to more directly measure relationships between brain state, mood, and cognitive performance. Future studies with a larger sample size should also enable modeling with a training set and a test set to generate more precise statistical estimates of mood symptom-behavior relationships.

## Conclusion

We identified differential patterns of mood-behavior relationships in younger vs. older online workers, with younger participants reporting high rates of anxiety and depression symptoms. These mood symptoms related to dissociable patterns of cognitive dysfunction: faster/inaccurate for those reporting higher anxiety, and slower/inaccurate for those reporting higher depression symptoms. These patterns differed by age, with older adults showing relatively preserved cognitive performance (slightly slower than younger participants, as hypothesized, but not differing on accuracy), and a mildly beneficial effect of subclinical anxiety and depression symptoms. There were no significant differences in average depression and anxiety symptoms compared to an age-matched pre-COVID reference sample (14). These results suggest a stable but concerning relationship between high mental health burden and worse cognitive function in younger adults working online. Increased access to mental health services in this group could potentially mitigate the impacts of the pandemic on wellbeing, workforce participation, and long-term health outcomes. Telehealth or smartphone-based mental health services may be particularly relevant for younger online workers.

## Data availability statement

The raw data supporting the conclusions of this article will be made available by the authors, without undue reservation.

## Ethics statement

The studies involving human participants were reviewed and approved by the Stanford University IRB. The patients/participants provided their written informed consent to participate in this study.

## Author contributions

CM-F: study design, implementation, data analysis, data visualization, and manuscript preparation. HS: data analysis, data visualization, and manuscript preparation. NH: study implementation and data collection. SN: study design and data collection. TH and RO’H: manuscript preparation. SB: manuscript preparation and data analysis. All authors contributed to the article and approved the submitted version.
